# Identification of Upper and Lower Level Yield Strength in Materials

**DOI:** 10.3390/ma10090982

**Published:** 2017-08-23

**Authors:** Jan Valíček, Marta Harničárová, Ivan Kopal, Zuzana Palková, Milena Kušnerová, Anton Panda, Vladimír Šepelák

**Affiliations:** 1 Institute of Physics, Faculty of Mining and Geology, Vysoká škola báňská—Technical University of Ostrava, 17. listopadu 15, 708 33 Ostrava, Czech Republic; marta.harnicarova@vsb.cz (M.H.); ivan.kopal@vsb.cz (I.K.); milena.kusnerova@vsb.cz (M.K.); vladimir.sepelak@kit.edu (V.S.); 2 Regional Materials Science and Technology Centre, Vysoká škola báňská—Technical University of Ostrava, 17. listopadu 15, 708 33 Ostrava, Czech Republic; 3 Technical Faculty, Slovak University of Agriculture in Nitra, Tr. A. Hlinku 2, 949 76 Nitra, Slovakia; zuzana.palkova@uniag.sk; 4 Department of Numerical Methods and Computing Modelling, Faculty of Industrial Technologies in Púchov, Alexander Dubček University of Trenčín, Ivana Krasku 491/30, 020 01 Púchov, Slovakia; 5 Faculty of Manufacturing Technologies with a Seat in Prešov, Technical University of Kosice, Bayerova Street 1, 080 01 Prešov, Slovakia; anton.panda@tuke.sk; 6 Institute of Nanotechnology, Karlsruhe Institute of Technology, Hermann-von-Helmholtz-Platz 1, 76344 Eggenstein-Leopoldshafen, Germany

**Keywords:** water jet technology, materials parameters, yield point, surface, deformation

## Abstract

This work evaluates the possibility of identifying mechanical parameters, especially upper and lower yield points, by the analytical processing of specific elements of the topography of surfaces generated with abrasive waterjet technology. We developed a new system of equations, which are connected with each other in such a way that the result of a calculation is a comprehensive mathematical–physical model, which describes numerically as well as graphically the deformation process of material cutting using an abrasive waterjet. The results of our model have been successfully checked against those obtained by means of a tensile test. The main prospect for future applications of the method presented in this article concerns the identification of mechanical parameters associated with the prediction of material behavior. The findings of this study can contribute to a more detailed understanding of the relationships: material properties—tool properties—deformation properties.

## 1. Introduction

The increasing number of industrial applications for abrasive water jet cutting technology entails many questions about possibilities for the process’s improvement. A basic understanding of the mechanism of the material’s disintegration by a water jet is required for the improvement of this technology. This technology represents a high energy beam as a cylindrical stream or jet of high-velocity water. The area of abrasive water jet cutting is characterised by the cut trace retardation and roughness formed in the course of abrasive water jet cutting. The abrasive water jet cutting technology and the accompanying phenomena occurring in the process of material cutting have been the subject of many research activities aimed at optimizing the technological parameters affecting the quality of a cut. From the literature [1,2,3,4,5,6,7,8,9,10,11,12,13] dealing with the problems of the quality of the cut wall surfaces in relation to the setting of a technological process in the case of abrasive water jet cutting technology, it follows that study of the surface quality and development of a topography function during cutting present a very questionable issue at present. Many authors use statistical, empirical, and mathematical models for the quantification of the various influences of technological parameters of the process of abrasive water jet cutting [1,2,3,4,5,6,7,8,9,10,11,12,13]. Based on a literature search, it can be stated that most of them are based on a regression analysis. It can be critically stated that the majority of the existing procedures are, from the point of view of use in operating practice, problematical, namely due to the problematical determination of a series of used constants, due to the concretization of inputs to derived relations, etc.

Most standardized methods for the determination of material parameters are based on the use of test specimens with a well-defined standardized geometry and loading. The development of effective whole-field measurement techniques has disclosed a new area of testing procedures to identify the constitutive equations of materials. Standard testing methods are used to infer the values of constitutive parameters with the help of simple exact solutions under an assumption of homogeneous strain and stress fields in the zone of interest, and full-field measurements allow considerably greater flexibility. In the literature, one can find a lot of methods for solving this problem, namely the finite element model updating model, the constitutive equation gap method, the virtual fields method, the equilibrium gap method, and the reciprocity gap method [14]. Only a limited number of parameters can be determined for each test with this classical approach. This has led the authors to consider an alternative approach directly based on the measurement of surface roughness.

On the basis of the analysis of the detected values of a surface roughness parameter *Ra*, we are able to evaluate the mechanical parameters of material in the elastic–plastic range. Our own solution is based on the analysis of the physical–mechanical and deformation equilibrium. The equilibrium state was determined for materials in general at the depth level of *h*_0_ (mm), thus identifying the position of the neutral plane in the dividing section, as well as the stress–strain state of the material. An important factor was a determination of the relationship of the ongoing process in cross-section to the Young’s modulus of the elasticity of the material *E_mat_*. With further development over the whole deformation length, i.e., the cut depth, it is possible to cover the individual main stress–strain limits. This concerns the limit of elasticity *Rel*, the yield strength *Re*, the strength limit *Rm*, and the rupture limit *R_fr_*. In this way, we obtain an analytical construction of the deformability diagram *σ_def_–h* or *σ_def_*–*ε*. It is possible to obtain analogically the values of these limits on the curve *σ_def_* (engineering stress) as a function of the curvature angle of the cut trace *δ*. The derivation of the necessary equations for the analytical description was preceded by the study of surfaces on different materials, which were generated by differently chosen technological parameters of the technology.

## 2. New Approach to the Solution

A theoretical basis for the derivation of the topography function for selected main variables consists in the use of stress–strain parameters of the cut material in conjunction with a solution for the mechanical equilibrium of the system: material properties—tool properties—deformation properties. We started with a description of the generated surfaces by expressing all of the surface geometry. Three widely recognized elements of the geometry of the cut wall surfaces at a given depth *h* can be identified:

1. The irregularities known as roughness *Ra* that often result from the cutting process.
2. Cut trace retardation *Y_ret_*.
3. The angle of curvature of the cut trace (deviation) *δ*.

**Figure 1:**
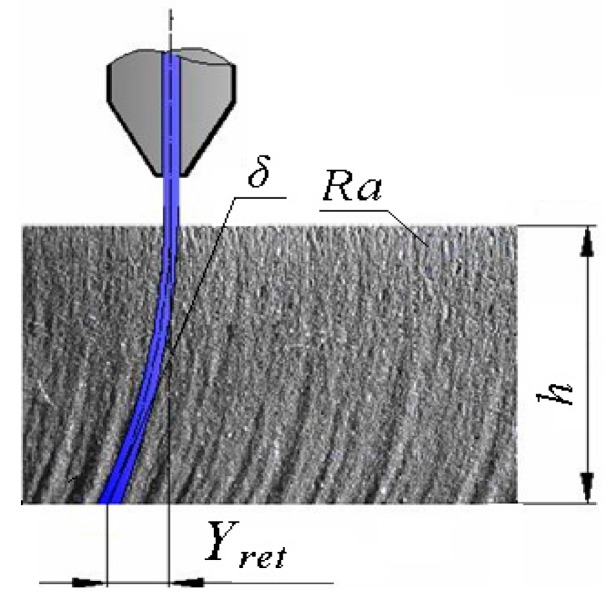
Geometrical parameters of the cut edge in the case of abrasive water jet cutting.

Figure 1 shows what is expected from the above-mentioned elements of the geometry. The depth of the jet’s penetration is also very important for our new approach.

The term “tool properties” can be replaced by the term “technology properties”. The whole set of properties, physical, mechanical, and technological, is closely connected with and affects the mechanism of the surface’s disintegration. A serious technological factor in material machining is the index of material machinability. This represents an indicator of suitability for use of specific technological parameters for material machining. In order to improve the properties of abrasive water jet cutting technology, it is necessary to introduce a mathematical approach to the assessment of material machinability limits and classes. For this reason, a new term “plasticity” of material *K_plmat_* is used. The parameter *K_plmat_* is based on the direct measurement of selected geometrical elements at any point on the surface of the cut wall. It is a comprehensive and empirical material parameter expressed in physical units [µm] that satisfies the Equation (1) below, and which is of high importance for other analyses of the process.

(1) $$K_{plmat}=\frac{Ra\cdot h}{Y_{ret}}=\frac{10^{12}}{E_{mat}^{2}}$$

Moreover, the parameter *K_plmat_* provides a direct connection to the elasticity–strength properties of the cut materials and to the laws of classical elasticity and strength, because a relation between the parameter and Young’s modulus in tension *E_mat_* in the following forms is also valid with sufficiently verified closeness. An important analytical factor is the determination of the equilibrium/neutral plane *h*_0_ in the cut produced by abrasive water jet cutting. It represents a case of the depth level in the cuts, where the tensile stresses and compressive stresses will be equalized. For *h* < *h*_0_, the tensile stress predominates; surface roughness is relatively low. For *h* > *h*_0_, the compressive stress increases and the roughness of the cut surface increases. It was found that in the course of cutting using an abrasive water jet tool, the tensile stress and the compressive stress were always equalized at the roughness values *Ra*_0_ = 3.7 μm, independently of the material, but at adequate depths of the neutral plane *h*_0_ = *h_Re_* that are different for different materials. Then, the depth of the neutral plane in the cut *h*_0_ = *h_Re_* must be adequate to these values, and the equation of mechanical equilibrium at the depth level of neutral plane *h*_0_ = *h_Re_* is defined as (2)

(2) $$\frac{Ra_{0}\cdot h_{0}}{Y_{ret0}-K_{plmat0}}=1.$$

The position *h*_0_ is also the position of *Re*, and we read the *Re* value from the *σ_def_*; therefore, *h*_0_ = *h_Re_*. At the general depth level the following is valid (3), where *K_plmat_* is a constant valid for all depth levels of the cut.

(3) $$\frac{Ra_{x}\cdot h_{x}}{Y_{retx}-K_{plmatx}}=1$$

Analytical processing of the main topographic surface parameters of the cut wall according to Figure 1 follows to the derivation of Equation (4) (roughness of cut trace at the depth), Equation (5) (appropriate depth of cut), Equation (6) (retardation of trace), Equation (7) (tangent of the angle of trace curvature), and Equation (8) (angle of trace curvature).

(4) $$Ra=\left(-10\right)\cdot \left(\frac{1-K_{plmat}}{K_{plmat}-h}\right)$$

(5) $$h_{Ra}=\frac{0.1\cdot Ra\cdot K_{plmat}}{\left(0.1\cdot Ra+1\right)},\;h=h_{Ra}$$

(6) $$Y_{ret}=\frac{Ra\cdot h}{K_{plmat}}$$

(7) $$tg\;\delta =\frac{Y_{ret}}{h}$$

(8) $$\delta =\frac{tg\left(\frac{Y_{ret}}{h}\right)\cdot 180}{3.14}$$

For this purpose, Equation (9) for radial roughness *Ra_d_* was modified. Equation (4) is empirical and it describes the basic topography function in the radial plane.

(9) $$Ra_{d}=10^{log\sqrt{\left({\left(log\left(h\right)\right)}^{2}+{\left(log\left(\frac{1}{Y_{ret}}\right)\right)}^{2}+Ra_{rad}^{2}\right)}}$$

Here we have:

(10) $$Ra_{rad}=Ra_{0}\cdot 10^{3}\cdot \frac{\sqrt{E_{retz}}}{E_{mat}},$$

(11) $$E_{retz}=E_{mat}\cdot \sqrt{\left(\frac{Ra\cdot h}{K_{plmat}}\right)}.$$

## 3. Experimental Setup

The abrasive water jet cutting system CNC WJ2020B-1Z-D (PTV Ltd., Hostivice, Czech Republic) was used in the experiment. This system consists of an intensifier pump connected to the cutting head. The cutting head is composed of a sapphire orifice, an abrasive water jet nozzle, and a mixing chamber. In order to investigate the influence of abrasive water jet cutting parameters on the depth of the cut and especially on the surface roughness, the factors selected for investigation are given in Table 1.

The preparation for the measurement and the version of implementation were the same for all materials. For cutting test samples of the length of 30 mm, small timber poles (with a length of 1000 mm and a square cross-section of 30 × 30 mm) were used. The surface profile was measured by a newly modified reflection method. The laser beam from the light source impinged the measured sample surface. The sample was moving at a constant speed perpendicularly to the line of sight. The incident beam was scattered by the surface irregularities of the surface towards the lens and passed through the slit, where it was recorded by a photodetector, by means of which the optical signal was transformed into an electrical signal. The slit plane was slightly shifted out of the plane of the focused image of the surface. This shift enabled the visualization of the phase differences of the light, which was scattered by the rough surface. The electrical signal was processed by a digital oscilloscope, and it was transmitted to a personal computer (PC). In such a way, the time dependencies corresponding to the surface roughness of the samples were obtained.

**Figure 2:**
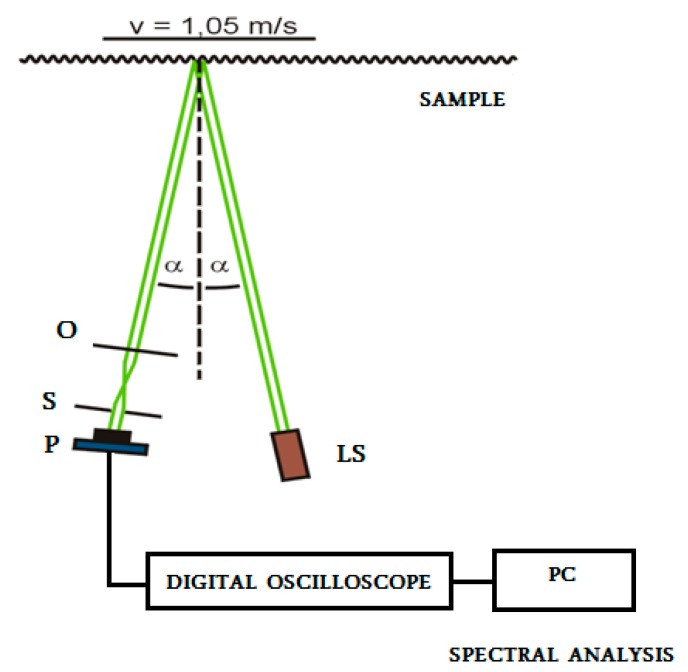
The arrangement of the experiment for surface roughness measurement using spectrum analysis.

The method is sensitive to qualitative differences in technological methods for the final machining of the surfaces (machined standards), i.e., machining by planing and front grinding. A comparison of the obtained results with standard values of the parameters *Ra* and *f_max_* (*f_max_* is the characteristic frequency of a vertical surface irregularity) was performed. The agreement was very good. The value of the correlation degree *r* was from 0.83 to 1.00. Figure 2 shows the basic principle of the measurement, where: P is the photodetector, S is the slit, O is the lens, LS is the light source, *α* is the angle between the light beam and normal line to surface, and v is the speed.

On the basis of the analysis of the measurement results and the results of analytical studies, an interactive mathematical–physical model can be created and an exact method of acquiring the equivalents of the mechanical parameters from the topography of surfaces generated by abrasive water jet cutting can be determined.

## 4. Testing Methodology, Measured and Comparative Analytical Data, and Graphs

The method of exact yield point identification is shown for the example of the steel grades S335J0, 15230QT, and AISI 316/316L, and for the aluminium alloy AW6082T6. The preparation of the samples, shaping of the test specimens, laboratory measurement of mechanical parameters, and bursting tests were carried out in the accredited testing laboratory of mechanical properties of company VÚHŽ in Dobrá, Czech Republic. Standardized test pieces of the circular cross-section were produced from the supplied materials in accordance with ČSN EN ISO 6892-1, with the following dimensions: *d_o_* = 8 mm, *l_o_* = 35 mm, and *l_c_* = 45 mm. The tensile tests were performed in accordance with the above standard using a verified electromechanical tensile testing machine with a maximum load of 100 kN, equipped with an ISO 7800 precision strain gauge. The applied stress rate was 15 MPa/s in the elastic deformation area, and also in the area of plastic deformation. The test was controlled by a strain rate of 0.0024 s^−1^. Each evaluated series included a total of 10 test pieces. The selected materials show very different values of basic mechanical parameters. These parameters characterize the shape of the curves *σ_def_*= f (*A*), or *σ_def_*= f (*ε*) in the deformability diagrams. They are presented in Table 2, Table 3, Table 4 and Table 5. The shape of the analytical curves for the theoretical equivalents of stress *σ_def_* given by the tensile test can be calculated as the function *σ_rzxE_*= f (*Ra*, *E_mat_*, cos *δ*) or as the function *σ_radx_*_=_ f (*Ra_d_*_,_ *E_mat_*, cos *δ*) according to (12)–(14):

(12) $$\sigma_{rzxE}=10^{-3}\cdot \frac{Ra\cdot E_{mat}}{Ra_{0}\cdot \cos\delta },$$

(13) $$\sigma_{radx}=10^{-3}\cdot \frac{Ra\cdot E_{mat}}{Ra_{d0}\cdot \cos\delta },$$

(14) Rad0=Ra0⋅103⋅ReEmat.

The theoretical stresses *σ_rzxE_* and *σ_radx_* according to (12) and (13) are defined as components of the deformation stress *σ_def_* in the outer envelope of diagrams *σ_def_*–*h* or *σ_de_*_f_–*ε* resp. *σ_def_–A*. Analytical equivalents from the tensile test are for comparison given below.

**Figure 3:**
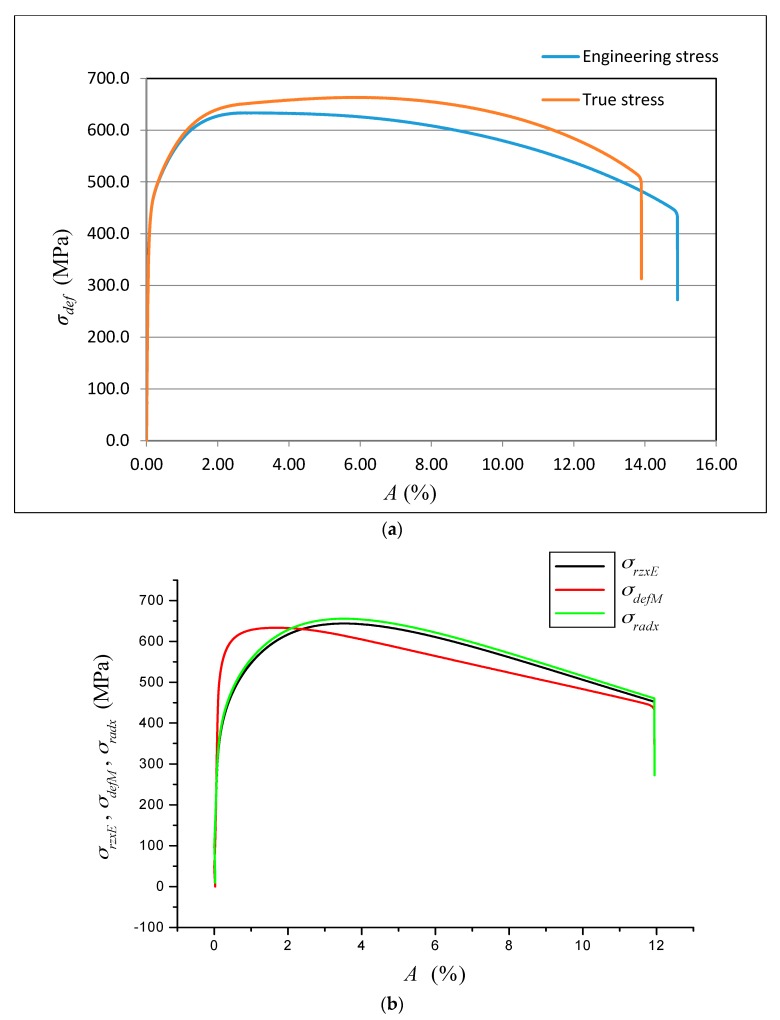

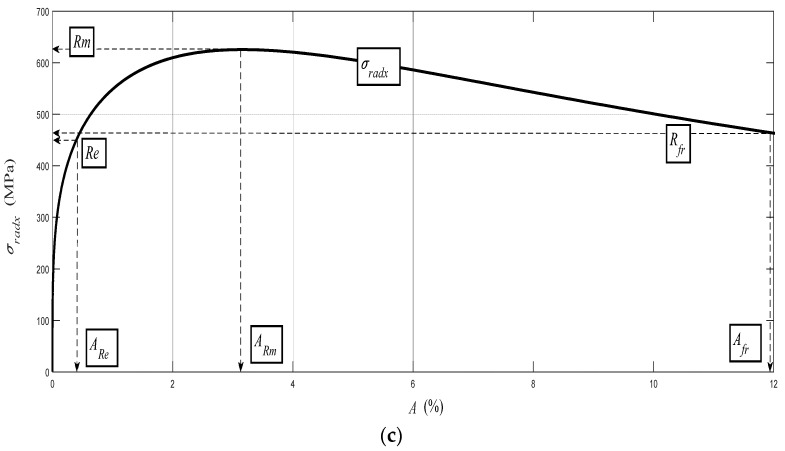
(**a**) Deformational diagram from the tensile test for the steel grade S355J0 for the dependence *σ_def_* = f (*A*); (**b**) Comparison of results (*σ_rzxE_*, *σ_defM_*, *σ_radx_*) = f (*A*) for the steel grade S355J0; Stresses *σ_rzxE_* and *σ_radx_* were derived analytically, and *σ_defM_* is obtained from the tensile test; (**c**) Analytical deformational diagram for the steel grade S355J0 for the dependence *σ_radx_* = f (*A*); comparing diagram.

**Figure 4:**
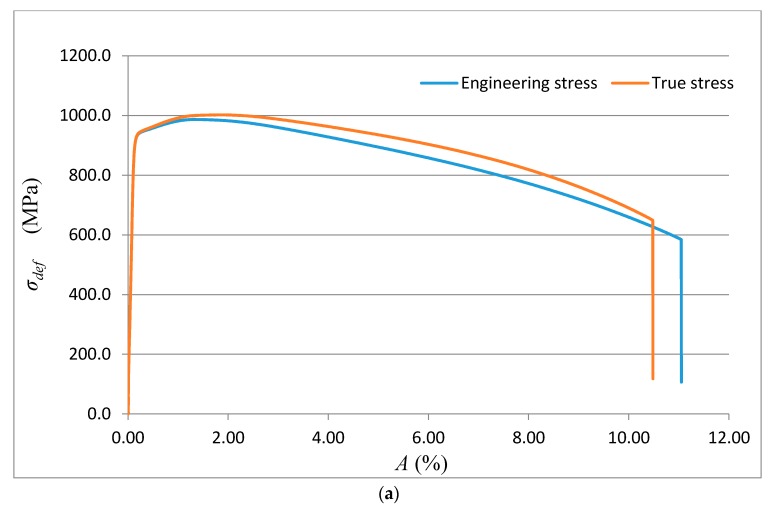

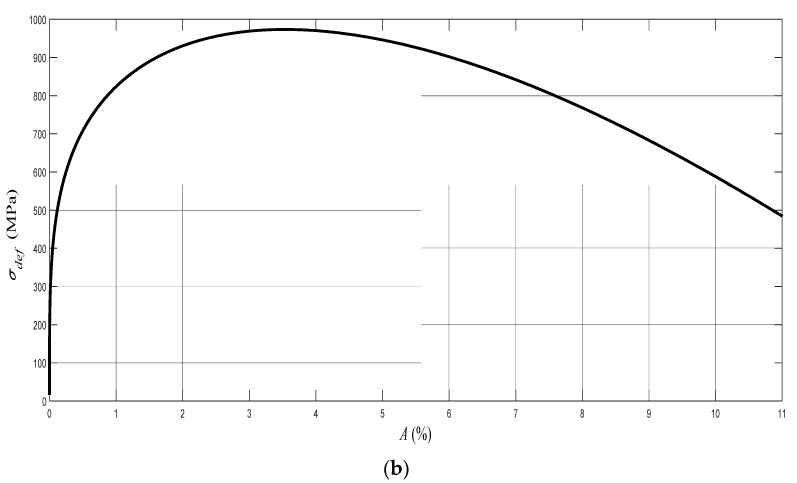
(**a**) Deformational diagram for the steel grade 15230QT for the dependence *σ_def_*= f (*A*) from tensile test; (**b**) Comparison diagram for the steel grade 15230QT; analytical construction of the dependence *σ_def_*= f (*A*).

**Figure 5:**
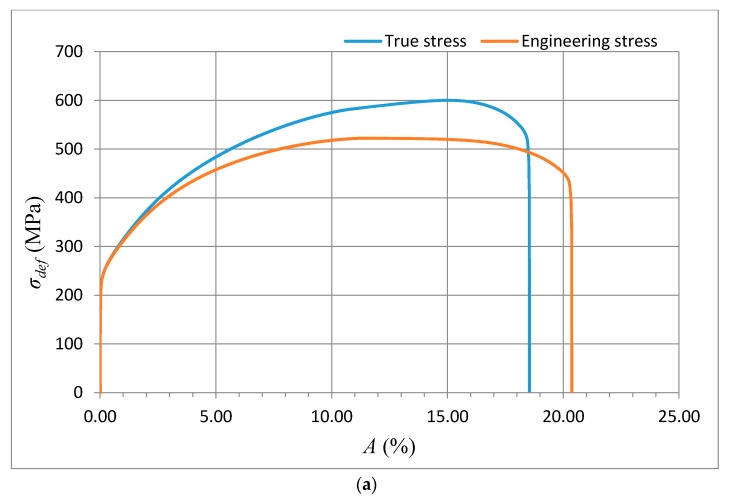

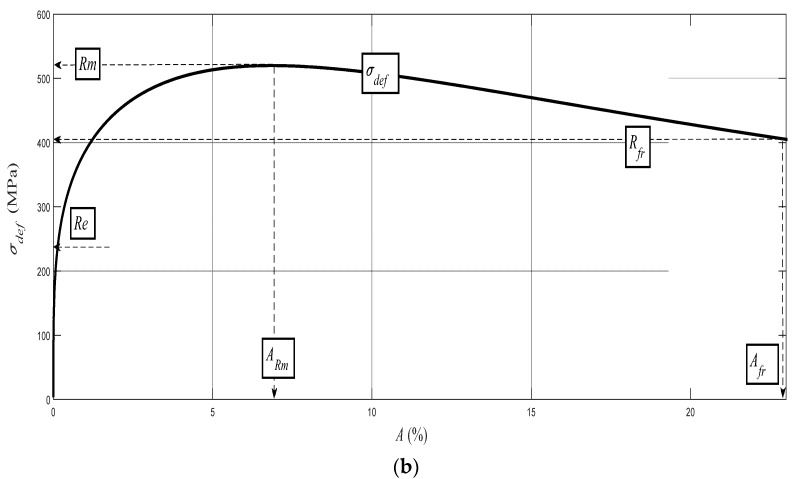
(**a**) Deformational diagram for the steel grade AISI 316/316L, for the dependence *σ_def_*= f (*A*) from tensile test; (**b**) Comparison diagram of the steel grade AISI 316/316L; analytical construction of dependence *σ_def_*= f (*A*).

**Figure 6:**
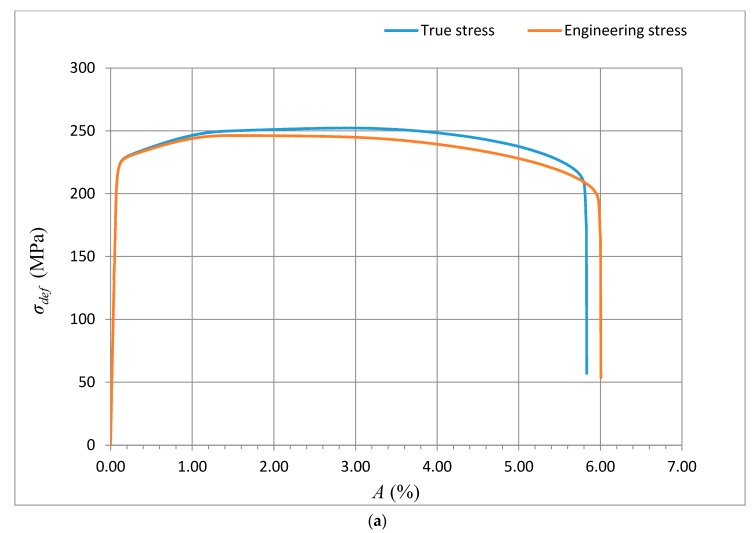

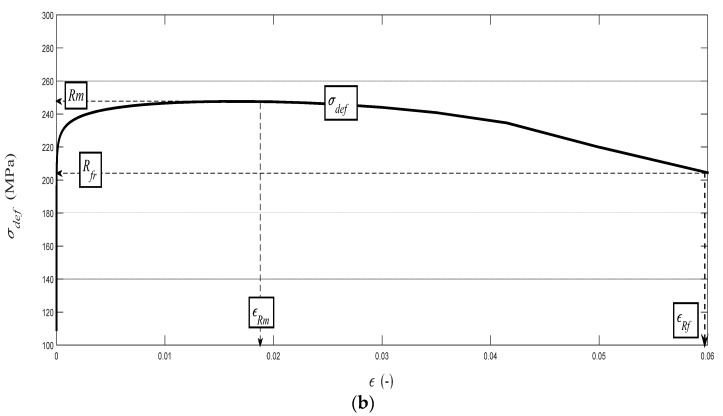
(**a**) Deformational diagram *σ_def_*= f (*A*) for the Al-alloy AW6083T6 from tensile test; (**b**) Analytical deformational diagram for AW6083T6, for the dependence *σ_def_*= f (*A*); comparison diagram.

The data obtained by the laboratory tests, as well as the comparison diagrams, are shown in Figure 3, Figure 4, Figure 5 and Figure 6.

The deformation stress *σ_defM_* measured by a testing laboratory is used to compare the tightness with the analytical results for the steel grade S355J0 through Equations (12)–(14) in the text. The outer envelopes of the theoretical values of stress *σ_rzxE_* (12) and *σ_radx_* (13) are the same. The theoretical stress values of both types are listed in the comparison for the following reason: *σ_rzxE_* is a function of (*E_mat_*, *Ra*, and *Ra*_0_), and thus the roughness *Ra* in the cut trace *σ_radx_* is a function of (*E_mat_*, *Ra_d_*_0_), where *Ra_d_*_0_ is the roughness across cut traces according to practice.

The plastic flow (hardening) looks different between the analytical and practical results. This is not surprising. The measured series for each material, according to the EN ISO 6892-1 standard [15], consists of a total of 10 measured samples. A certain difference compared to the average can be seen for each of them. This note also applies to the other tested materials in this article.

## 5. Identification of the Upper and Lower Yield Points

When a material is characterized by an abrupt drop of load at the yield point, both upper and lower yield stresses are available measures of elastic strength. The maximum or upper yield strength, although it appears to be advantageously well-defined, is rarely relied upon because the initiation of yield is extremely sensitive to test conditions and thus difficult to reproduce. Our solution provides an alternative to identification of the upper and lower yield points [16,17,18,19]. The analytical identification is based on the theory of determination of the intersection of the evolution of pressure components in the modular, stress, and deformation regions. The modular area is given by the value of the modulus of elasticity *E_mat_* of the investigated material. The pressure modular component is then given by the evolution of the *E_retz_* curve according to Equation (11). The tensile modular component is given by the evolution of the *E_ret_* curve according to Equation (15) below.

(15) $$E_{ret}=E_{mat}\cdot \sqrt{\left(\frac{K_{plmat}}{Ra\cdot h}\right)}.$$

The stress and deformed regions are resolved analogically. The stress region is given by the value of the yield point of the studied material and by the pressure component *σ_rz_* according to Equation (16) or the tensile component *σ_ret_* according to Equation (17).

(16) $$\sigma_{rz}=\sqrt{E_{retz}},$$

(17) $$\sigma_{ret}=\sqrt{E_{ret}}.$$

The deformation region is given by the roughness value *Ra*_0_ at the depth level of the so-called neutral plane *h_0_* of the studied material. The pressure component of the surface deformation *Ra_rz_* is determined by the Equation (18) and the tensile component *Ra_ret_* according to Equation (19). The intersection of the deformation components *Ra_rz_* and *Ra_ret_* sets the *Ra*_0_ value.

(18) $$Ra_{rz}=Ra_{0}\cdot \sqrt{\left(\frac{Ra\cdot h}{K_{plmat}}\right)},$$

(19) $$Ra_{ret}=Ra_{0}\cdot \sqrt{\left(\frac{K_{plmat}}{Ra\cdot h}\right)}.$$

**Figure 7:**
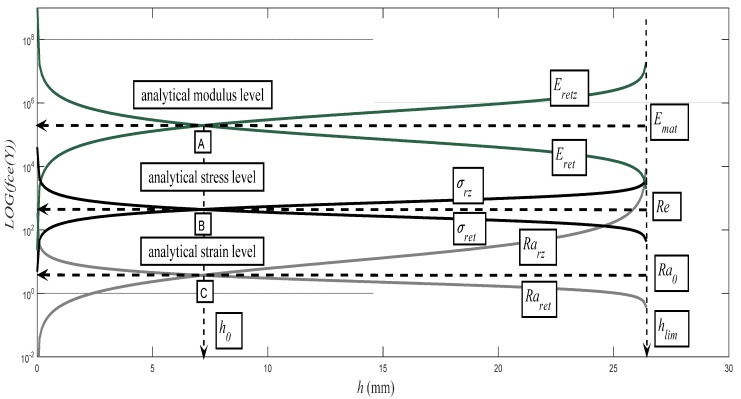
Steel S355J0, *E_mat_*= 194.5 GPa, analytical derivation of the position of the yield point *Re*, including the position of the neutral plane *h*_0_, the value *Re* being read from the *σ_rz_* curve at the level *h*_0_, i.e., *Re* = *σ_rz_*_0_; the yield point *Re* is defined by the intersection of the curves for the pressure and tensile stress components *σ_rz_* and *σ_ret_*. At this intersection at the neutral depth, *h*_0_ applies *Re* = *σ_rz_*_0_ = *σ_ret_*_0_. (Components *E_retz_*, *E_ret_* of analytical modulus level are marked green color, components of analytical stress level *σ_rz_*, *σ_ret_* are marked black color, components of analytical strain level *Ra_rz_*, *Ra_ret_* are marked gray color).

The evolutions of the thus calculated components for the material S355J0, *E_mat_* = 194.5 GPa, are illustrated as an example in Figure 7. The position of the yield strength *Re* is identical with the position of the neutral plane at the depth ho of the cut. The verified dependencies (*h*_0_, *Re*) = f (*h*, ε, *A*) are generally valid for materials at the intersection of the pressure and tensile components of the stress and deformation of the materials. This theory can be widely applied in various technologies for the exploitation of technical materials [8].

**Figure 8:**
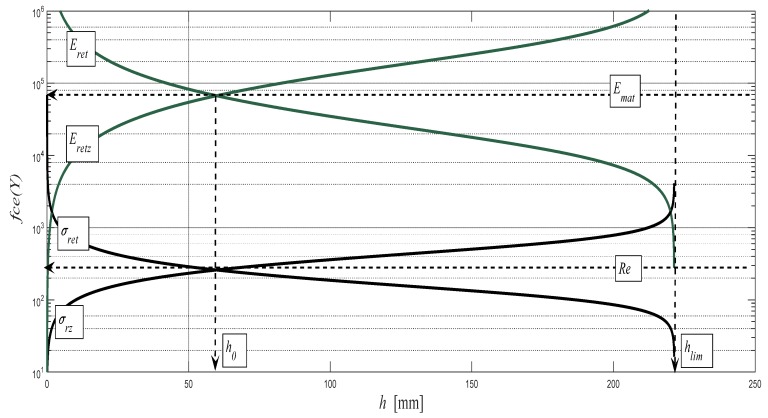
Theoretical identification of position in the diagram and of the values of dependence (*h*_0_, *Re*) = f (*h*), which can be analogically realized for the dependencies (*h*_0_, *Re*) = f (*ε*), or (*h*_0_, *Re*) = f (*A*) at tensile tests; material: aluminium alloy AW6082T6.

The theory finds its application mainly for such materials that do not show their yield strength position during classical laboratory bursting tests. These are namely structural materials with a low modulus of tensile elasticity *E_mat_* below 70 GPa. The structural steels should be trouble-free. However, the method of measurement and the technical design of tension testing machines does not allow for the accurate recording of *Re* and in more detail of *Re_U_* or *Re_L_*. Nevertheless, the correct value of *Re* is essential for designers, specifically for the appropriate dimensioning of structural materials and the structural components of machines, equipment, and buildings. This concerns ensuring the functionality, safety, and service life of structures. As to what concerns the selection of materials that are studied here, the most problematic is the aluminium alloy AW6082T6 with a relatively low modulus of also elasticity *E_mat_* = 67.2 GPa. We will, therefore, show using this material the practical use of the theoretical identification of *Re* and *h*_0_ values. The structure realized in practice according to this theory and equations is presented in the diagram in Figure 8. 

## 6. Yield Strength and the Degree of Safety of the Structural Materials

The choice of safety degree is a matter of empiricism gained by the operations and experience of the designer with the particular material and the type of mechanical stress on the structure. For the allowed stress, the relation is *σ_dt_ = σ_kt_*/*k*, where *σ_kt_* is flow stress and therefore *σ_kt_ = Re*. The degree of safety, or also the safety factor (Table 6), is here denoted by the letter *k*. For materials that do not show their yield strength in the measured values, the ultimate stress is used and the relation σ_dt_ = σ_pt_/k* is valid, where *σ_dt_* is the ultimate stress. In these cases, a higher safety factor k* is used (k* > *k*).

**Figure 9:**
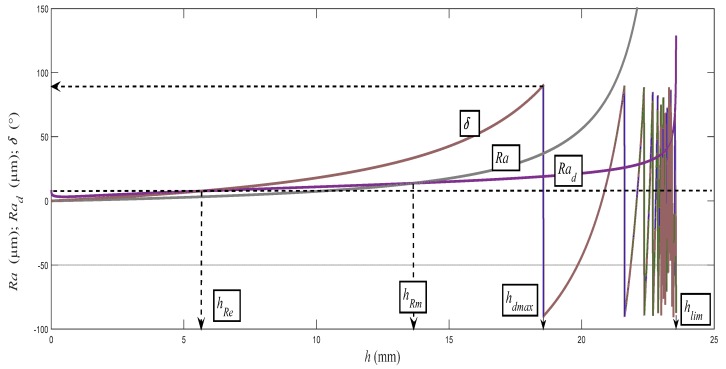
Topography functions (*Ra*, *Ra_d_*, *δ*) = f (*h*) for AISI 309 [8], *Ra* (gray color), *Ra_d_*(purple color), and *δ* (brick color).

The authors of the paper have been doing research on the topography of surfaces created by various cutting tools and technologies for several years already. They have dealt also with the static and dynamic stress on a material’s surface and on its structural elements. The basis for this was research using a flexible type of machining tool. It concerned mainly machining and cutting by a high-pressure abrasive beam and by laser. The flexible tool has its own specific advantages. It immediately responds to a change in a material’s resistance to disintegration. Any such change is immediately reflected in the topography of the disintegrated surface of the cut walls. It is possible to observe the transitions from the area of flexible to plastic deformations by the naked eye, using a magnifying glass or a microscope for a number of solid materials, and to estimate the position of the elastic limit and the yield strength. As the main elements of the geometry of the cut wall surfaces, the following parameters are proposed: surface roughness *Ra*, cut trace retardation *Y_ret_*, angle of curvature of cut trace (deviation) *δ*, and depth of the cut *h,* and potentially the thickness of the sample being cut (Figure 1). On the basis of the above-mentioned reasons, these basic geometric or topographic parameters occur as the main variables in almost all equations derived by us. It can be often admitted that they complement the mathematical solution procedures concerning classical elasticity and strength. The typical distribution of the topography functions according to the above-mentioned relation (9) is shown in Figure 9. The topographic elements of roughness *Ra*, *Ra_d_*, and the angle of curvature of the cut trace *δ*, generated with a flexible tool, as well as the angle-δ-determined delay of the cut trace *Y_ret_* against the radial plane according to the depth of cut *h* were well- and accurately measurable directly on the cutting wall of the test specimens. The topographic elements are basic attributes of the surfaces used to derive the so-called topographic function. Figure 9 presents their behaviors according to the instantaneous depth of cut *h* in relation to their analytical descriptions within the topographic function in its default form (*Ra*, *Ra_d_*, *δ*, ... *Y_ret_*) = f (*h*). The instantaneous deformation of the surfaces described by means of the basic topographic elements is induced by instantaneous deformation stress *σ_def_* at the point of direct contact of the disintegrating tool with the material. It also implies a unique opportunity to use topographic functions according to their default form to derive the behaviour of stress by the depth *h*, and thus the function *σ_def_* = f (*h*) or *σ_def_* = f (*ε*).

The existence of the upper and lower yield points is often discussed and this issue has not been clarified yet. In plastic, less strong materials, it is difficult to identify the position of the yield point from tensile tests. The determination of the yield point is conventional, and, for simplicity, the yield point is taken at 0.2% of ductility. The relation (9) is modified to (20) in order to emphasize the condition at a depth level of the neutral plane *h*_0_ = *h_Re_* and in the area of the yield point. In this conception and in accordance with the above-described conception of the mechanism of surface generation, a transition of deformation and dislocations from one creep plane to another can be justifiably considered at the yield point and at the level of the neutral plane *h*_0_ = *h_Re_*, where the tensile component is in balance with the compressive component of stress. By a sufficiently accurate measurement of roughness, this transition can be found also on the amplitudes of the local roughness directly on the machined surface. This local roughness can be designated as *Ra_q_* and analytically calculated according to a newly modified Equation (20). The development of the local stress *σ_rzq_* according to the relation (21) is related to the development of the local roughness in the yield point region.

(20) $$Ra_{q}=Ra_{0}\cdot \left(\left(\log{\left(h\right)}^{2}+\log{\left({\left(h\cdot tg\delta \right)}^{-1}\right)}^{0.25}\right)\right),$$

(21) $$\sigma_{rzq}=10^{-3}\cdot E_{mat}\cdot \frac{Ra_{q}}{Ra_{0}}\cdot \cos\delta^{h_{0}},$$

or in a form of the generalized *Hooke's law*

(22) $$\sigma_{rzq}=\varepsilon_{rzq}\cdot E_{mat},$$

(23) $$\varepsilon =\varepsilon_{rzq}=10^{-3}\cdot \frac{Ra_{q}}{Ra_{0}}\cdot \cos\delta^{h_{0}}.$$

The *σ_c_* function simulates stress in the material core, it means the permanent strength of the material, and it determines ductility on the envelope of the stress–strain curves.

(24) $$\sigma_{c}=\frac{E_{mat}^{0.5}}{K_{plmat}}\cdot h.$$

## 7. Identification of Parameters of the Upper and Lower Yield Strength

**Figure 10:**
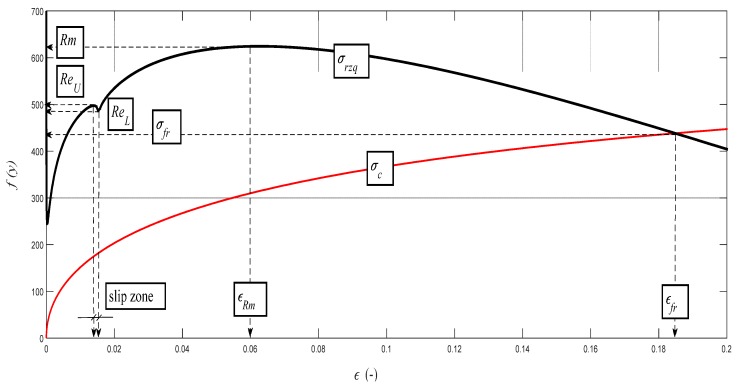
Analytical deformability diagram for the steel grade S355J0 for the dependence *σ_rzq_*= f (*ε*); detail for *Re_U_* and *Re_L_*. *σ_rzq_*(black color) and *σ_c_* (red color).

**Figure 11:**
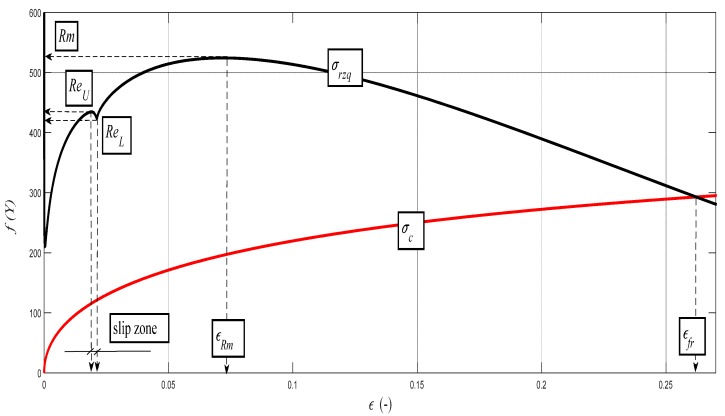
Analytical deformability diagram for the steel grade AISI 316/316L for the dependence *σ_rzq_*= f (*ε*); detail for *Re_U_* and *Re_L_*. *σ_rzq_*(black color) and *σ_c_* (red color).

**Figure 12:**
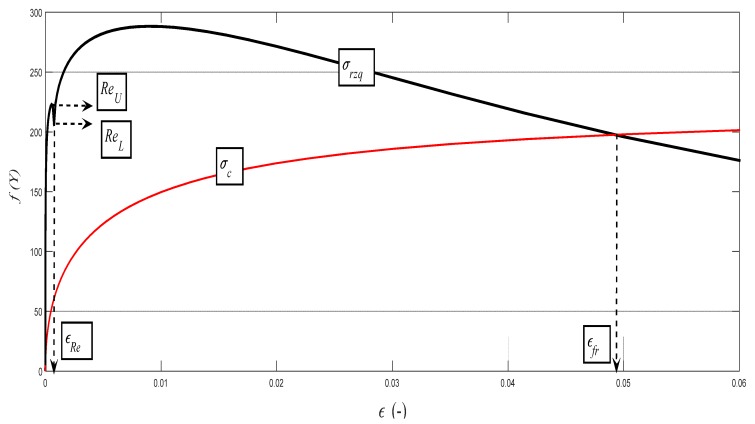
Analytical deformability diagram for the aluminium alloy AW6082T6 for the dependence *σ_rzq_* = f (*ε*); detail for *Re_U_* and *Re_L_*. *σ_rzq_*(black color) and *σ_c_* (red color).

**Figure 13:**
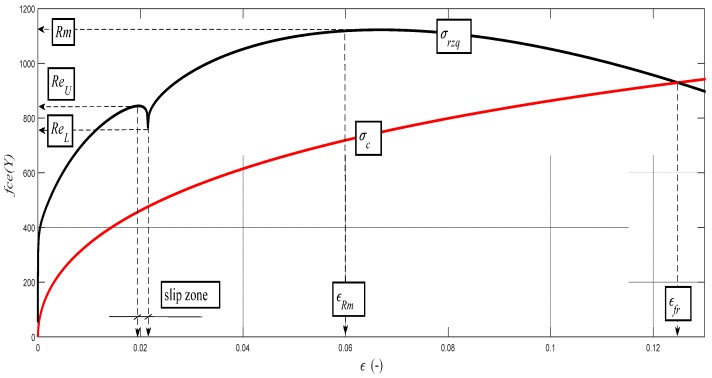
Analytical deformability diagram for the steel grade 15230QT for the dependence *σ_rzq_* = f (*ε*); detail for *Re_U_* and *Re_L_*. *σ_rzq_*(black color) and *σ_c_* (red color).

**Figure 14:**
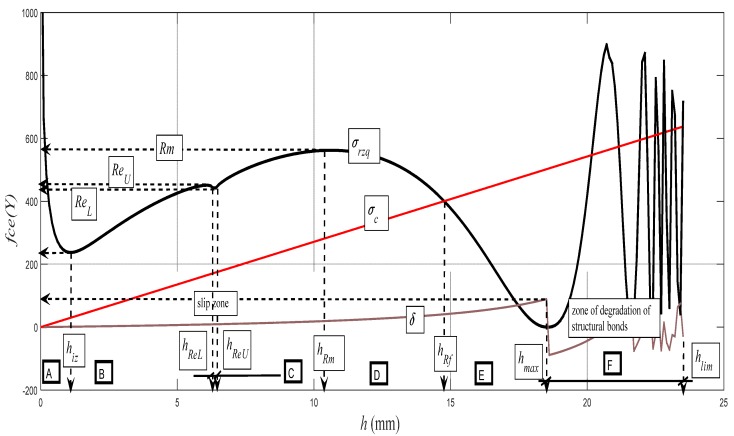
Entire development of the deformation stress for the steel grade AISI 309 in the case of dependence *σ_rzq_* = f (*h*). *σ_rzq_*(black color), *σ_c_* (red color), and δ (brick color).

Examples of diagrams with the analytical identification of the parameters of the upper and lower yield strength are shown in Figure 10, Figure 11, Figure 12, Figure 13 and Figure 14. The identification of the upper and lower yield strength is performed by the above given Equations (22)–(24) for parameters *σ_rzq_*, *ε,* and *σ_c_*. A verification of this method can be carried out in four basic ways: (a) checking by the results of tensile tests; (b) checking by material data sheets; (c) checking by tables; and (d) checking by the roughness measurements of *Ra* and *Ra_d_* on cuts using flexible cutting tools of abrasive waterjet.

The graph in Figure 14 represents the backward reconstruction of the cut and it expresses the theoretical dependence of the distribution of the deformation stress *σ_rzq_* = f (*h*) on the entire cut depth. Here, in addition to the positions of the principal stress–strain limits, qualitative changes in the character of distribution can be observed as well, namely according to the specific deformation zones A to F, which can be defined as follows:

- A zone: defined by a depth range 0–*h_iz_*, including the high resistance of the surface nanolayer;
- B zone: defined by a depth range *h_iz_*–*h_ReU_*, elastic deformation zone;
- C zone: defined by a depth range *h_ReU_*–*h_Rm_*, elastic-plastic deformation zone;
- D zone: defined by a depth range *h_Rm_*–*h_Rf_*, zone of plastic flow;
- E zone: defined by a depth range *h_Rf_*–*h_max_*, zone of structural deformations; and
- F zone: defined by a depth range *h_max_*–*h_lim_*, zone of degradation of structural bonds.

The concept of the zonality of an instantaneous stress–strain state and the consistency of the structure of the material that is subjected to external deformation stress is known from the field of material engineering. The authors wanted only to show that the zonality analogical from the physico-mechanical and topographical points of view can be measured as well as interpreted in this concept on the cuts carried out with flexible cutting or disintegrating tools. Zonality is reflected appropriately when using three-dimensional (3D) projection. Three-dimensional (3D) projection is gained as readings on modern profile meters. It is also possible to use the analytical method proposed by the authors to model satisfactorily the surface topography in 3D on a PC. For control and correlation, simple and expressive measurements of cutting walls by ultrasound can be used as well. In Figure 14, there is also very interesting zone A, where a range of valuable information can be analyzed up to surface nanolayers. A sharp increase in stress can be seen in the material and in the tool, while the roughness increases and the grain size decreases to the nanoscale. This A zone is called the initiation zone and indicates the high strength of the surface layer in nanoscale.

## 8. Discussion of the Concept of Solution and Results

Each of the materials has a certain deformation capacity of its surface layer and a certain deformation length of the cut *K_plmat_* up to the limit depth hlim, where the structure of the material is destroyed. The cut passes at a certain depth through a so-called neutral plane *h_0_*, which is specific for each material. At the neutral level, tensile and compressive stresses are compensated for, and their equilibrium intersection identifies the depth position *h*_0_ as well as the amplitude value of the yield strength *Re*, including shaping *Re_U_* and *Re_L_*.

It is analogous to the case of the stress of beams. For some solid materials, the change in the roughness *Ra_d_* on the crossing through the plane *h_0_* is apparent even to the naked eye. The topographic elements of roughness *Ra* and *Ra_d_*, and the angle of curvature of the cut trace *δ*, generated with a flexible tool, as well as the angle-*δ*-determined delay of the cut trace *Y_ret_* against the radial plane according to the depth of cut *h*, were well- and accurately measurable directly on the cutting wall of the test specimens. The topographic elements are basic attributes of the surfaces used to derive the so-called topographic function. In Figure 9, the authors wanted to present their behaviours according to the instantaneous depth of cut h in relation to their analytical descriptions within the topographic function in the default form (*Ra*, *Ra_d_*, *δ*, ... *Y_ret_*) = f (*h*). The instantaneous deformation of surfaces described by means of the basic topographic elements is induced by instantaneous deformation stress *σ_def_* at the point of direct contact of the disintegrating tool with the material. It also implies a unique opportunity to use topographic functions according to their default form to derive the behaviour of stress by the depth *h*, and thus the function *σ_def_* = f (*h*) or *σ_def_* = f (ε). The concept of the zonality of an instantaneous stress–strain state and the consistency of the structure of the material that is subjected to external deformation stress is known from the field of material engineering. The authors wanted only to show that the zonality analogical from physico-mechanical and topographical points of view can be measured as well as interpreted in this concept on the cuts carried out with flexible cutting or disintegrating tools. Zonality is reflected appropriately when using 3D projection. In Figure 14, there is also very interesting zone A, where a range of valuable information can be analyzed up to surface nanolayers. A sharp increase in stress in the material and in the tool can be seen, while the roughness increases and the grain size decreases to the nanoscale. This A zone is called the initiation zone and indicates the high strength of the surface layer in nanoscale. We are currently dealing with this issue.

## 9. Conclusions

The submitted work presents the results and new findings obtained by the study of the topography of surfaces generated by the cutting of engineering materials by an abrasive water jet flexible tool. The processing of the new findings is designed to cover at least some items from the critical evaluation of the current state of knowledge of laws of technologies, especially the principles of the disintegration mechanism and the creation of new surfaces. Hence, using the possibility to prepare a proposal for a comprehensive optimization of the selection of the main parameters of the technological modes for abrasive water jet cutting, various types of materials were used in order to reach the required depths at the required quality and price of cuts, and in the concept of the presented work to define differences in stress–strain characteristics, or in the deformation of material subjected to external stresses. The work also points out the fact that the used method for the analytical description of the principles of the disintegration mechanism can be applied in the engineering exploitation of materials, in the engineering design of structures concerning the required safety, stability, and service life of structural works, structures, and machines. Insufficiently accurate values of the fundamental physical and elasticity–strength parameters of the structural materials used for the stability and structural calculations cause an uncertainty of the given characteristics with potentially serious consequences. The results of the work can be specified according to the obtained findings by the derivation of the following items:

- equilibrium of deformation functions of the surface topography;
- deformation capacity, plasticity coefficient *K_plmat_,* and their relations to *E_mat_*;
- equations for the elements of surface topography in the trace of cuts and in the radial plane;
- a method of solving the stress–strain functions according to the surface topography;
- partial influence of tension and compression branches of stress on the surface deformation;
- construction of equivalents of diagrams *σ*–*h* and *σ*–*ε* from the parameters of the cut; and
- a method of dealing with and use of interactive mathematical modeling of the process.

A sufficiently accurate determination of the actual engineering and permanent Young’s moduli, elastic limit, yield point, and ultimate strength is of the highest importance to the safety, stability, and life of constructions, structures, and machines. From this, serious substantial requirements for searching for new accurate procedures for material testing as well as for new knowledge of theoretical and applied elasticity, strength, and mechanics follow.

The proposed concepts and ways of solution increase the quality and quantity of information and knowledge about very important physical and mechanical parameters of structural materials. In particular, this includes, in their exact analytical and graphical forms, the possibility for creating the necessary algorithms for the mathematical modeling of structural elements and entire structures. For the needs of designers and engineers, the desired physical and mechanical data are not always available for the purposes of proportioning load-bearing structures. Quite often, dangerous accidents happen to large buildings. Direct measurements of the required parameters are time consuming and expensive. Moreover, they still have a number of technical constraints. In this respect, analytical solutions have greater flexibility, and a greater volume of information for science and practice.

## Figures and Tables

**Table 1:** Technology factors.

Column	Technology Factors	Unit	Symbol	Value
1	Liquid pressure	MPa	*p*	300
2	Water orifice diameter	Mm	*Dv*_0_	0.25
3	Abrasive nozzle diameter	Mm	*d_abro_*	0.75
4	Abrasive nozzle length	Mm	*l_a_*	76
5	Abrasive mass flow rate	g/min	*m_a_*	250
6	Nozzle-surface distance	Mm	*L*	2
7	Traverse speed	mm/min	*v_popt_*	50, 100, 150, and 200
8	Abrasive size	MESH	-	80
9	Abrasive material	-	-	Barton garnet

**Table 2:** Mechanical parameters for S355J0 where *Z* is a contraction.

*E_mat_* (MPa)	*Re* (MPa)	*Rm* (MPa)	*A* (%)	*Z* (%)
194,500	450	635	15	64

**Table 3:** Mechanical parameters for 15230QT, where *Z* is a contraction.

*E_mat_* (MPa)	*Re* (MPa)	*Rm* (MPa)	*A* (%)	*Z* (%)
309,000	928	987	11	60

**Table 4:** Mechanical properties of the evaluated set AISI 316/316L.

*E_mat_* (MPa)	*Re* (MPa)	*Rm* (MPa)	*A* (%)	*Z* (%)
190,000	229	522	22.9	60.2

**Table 5:** Mechanical properties of the evaluated set AW6082T6.

*E_mat_* (MPa)	*Re* (MPa)	*Rm* (MPa)	*A* (%)	*Z* (%)
67,200	224	246	6	33

**Table 6:** Degree of safety for different materials.

Material	*k*, k*
Steel	*k* = 1.2–2
Quenched steel	k* = 2.5–4
Grey cast iron	k* = 4–5
Cast aluminium	k* = 8–10
Wood	k* = 6–12
Concrete	k* = 4–8
